# Evidence-based medicine: How evident is it ?

**DOI:** 10.4103/0974-2069.74034

**Published:** 2010

**Authors:** Bharat Dalvi

Evidence based medicine (EBM) has become the *mantra* of contemporary clinical practice. Journals, meetings, seminars, and workshops emphasize its importance and strongly advocate its use. From pharmacological treatment of heart failure to device closure of atrial septal defects to role of hybrid procedures for hypoplastic left heart syndrome or surgical intervention in Kawasaki disease, it is EBM all the way. For the want of anything better, we are left with very little choice but follow this model of EBM. However, one wonders as to how strong this “evidence” is when the results of prospective, randomized controlled trials (RCTs) considered gold standard for EBM, are used in our day-to-day clinical practice.

Most clinical practice in pediatric cardiology and cardiac surgery is based on results of observational studies and on the opinions of experts, as there are ethical and other logistic issues in conducting RCTs in children.[1] Often we take the results of trials conducted in adult populations and rightly or wrongly, extrapolate them to children and even newborns despite knowing that they are not “miniaturized adults.” It is, therefore, essential that we understand the limitations of RCTs or observational studies, which guide patient care in our specialties.

Most multicentric RCTs are conducted in populations of significant diversity. They are, however, so designed that the baseline parameters tend to match in the treatment group vis-a-vis the controls. This is absolutely essential in order to compare outcomes between groups. Let us take an example of the three, not so recent trials, which continue to dictate our current practice of using angiotensin converting enzyme inhibitors in cardiac failure. All of them consider risk factors like smoking, hypertension (HT) and diabetes mellitus (DM) as discrete variables with dichotomous distribution.[2–4] Is not that fallacious when we look at the real life scenario? Can someone who is smoking 40 cigarettes for 40 years be equated with someone who is smoking 10 cigarettes for 5 years. Similarly, a hypertensive with a baseline blood pressure of 210/130 mmHg requiring three antihypertensives to control his pressure cannot be obviously put in the same basket as another with a baseline pressure of 150/100 mmHg needing just one drug to remain normotensive. The same is true for a diabetic controlled with a single oral hypoglycemic agent, versus one who needs 40 units of insulin twice a day. In a way these are continuous variables but defining them in that fashion does not appear to be simple. This variability in the two groups, which apparently look similar, can significantly affect outcome events and is ignored in most RCTs.[2–4] Variability is further complicated by the presence or absence of end organ damage in each of these patient subgroups. How do we factor that into our current models of conducting trials?

Another drawback of some of these major trials[2–4] is the use of univariate methods rather than multiple logistic regression for comparing baseline characteristics. Multiple univariate comparisons alone may not reveal baseline differences among the treatment groups[5] and although, the process of randomization is known to negate this problem to some extent, these differences could vitiate the overall results. This may be one of the reasons why two trials studying effect of the same intervention may not produce same or even similar results.

In order to keep the population uniform and not subject patients at “high risk” to trial protocols, a number of inclusion and exclusion criteria are proposed in each of these trials. Unfortunately, in the real life scenario we tend to rely on these results without considering whether or not the patient in question fulfills all these criteria. What happens if the patient fulfills only two out of four inclusion and three out of five exclusion criteria? This could produce discrepant responses in an individual patient when compared to those in the trial.

Most RCTs are designed to address a larger question, e.g., does thrombolysis increase survival in patients with AMI.[6,7] Due to inherent problems associated with subgroup analysis[5], it is impossible to know which of the subgroups did not benefit from the intervention. In the absence of such information, subjecting all the patients to that intervention which has a potential to produce life-threatening complications, makes the decision in real life quite difficult. How do we practice EBM under these circumstances?

Let me digress a little to observational studies in our specialties. While assessing incremental risk factors for an unfavorable outcome, we tend to describe patient variables, disease variables, procedure-related variables but rarely do we describe operator(s) related human variables which in my opinion impact outcome the most. This is compounded by the fact that there are just too many people involved in taking care of these babies– surgeons, anesthetists, perfusionists, operating room nurses, ICU staff, physiotherapists and many others. Each one has different experience, expertise, training, temperament, team spirit and a number of other physiological/psychological variables (including duration of sleep he/she had the previous night, anxiety in his/her personal or professional life and many others) which will impact their performance and thereby the net outcome. How do we account for these critical variables?

Lastly, making rules in a biological system is quite difficult due to the number of linear and nonlinear variables involved in a living organism. RCTs are certainly not an exception. In most RCTs we rely upon a few thousand (or even a few hundred) patients to make rules. Logistics and economics do not permit us to study millions of patients under a controlled environment thus making RCTs far less reproducible and dependable, compared to laws of physics or chemistry, which more often than not, take into consideration the behavior of a few million or billion atoms or molecules.[8] With the astronomical numbers involved in the study of atomic or molecular macrosystems, the randomness in behavior tends to give way to some form of orderliness. Take for example the law of paramagnetism or the law of diffusion. Both are based on the principle of atomic statistics, and therefore, are imprecise (approximate) when applied to a few hundred atoms. Their precision and reproducibility is strictly based on the large number of atoms in the system under observation.[8]

RCTs will continue to remain the gold standard in the foreseeable future for the want of anything better but in their current form they will be far from perfect in formulating rules. Therefore, common sense and logic should be exercised, while extrapolating data from RCTs to clinical practice. The so-called EBM is yet to prove itself in that sense.
